# Examining the Efficacy of a Very-Low-Carbohydrate Ketogenic Diet on Cardiovascular Health in Adults with Mildly Elevated Low-Density Lipoprotein Cholesterol in an Open-Label Pilot Study

**DOI:** 10.1089/met.2021.0042

**Published:** 2022-03-15

**Authors:** Nikolaos Tzenios, Erin D. Lewis, David C. Crowley, Mohamad Chahine, Malkanthi Evans

**Affiliations:** ^1^ Public Health and Medical Research, Charisma University, Grace Bay, Turks and Caicos Islands.; ^2^ Global Clinical Scholars Research Training Program, Department of Postgraduate Medical Education, Harvard Medical School, Boston, Massachusetts, USA.; ^3^ KGK Science, Inc., London, Ontario, Canada.; ^4^ Biological and Chemical Technology, International Medical Institute, Kursk State Medical University, Kursk, Russian Federation.

**Keywords:** ketogenic diet, cardiovascular disease, cholesterol, lipid profile, body composition, weight loss

## Abstract

**Background:**

The objective of this open-label pilot study was to investigate the efficacy of a very-low-carbohydrate ketogenic diet (VLCKD), known as Nic's Ketogenic Diet, for 140 days on cardiometabolic markers in healthy adults with mildly elevated low-density lipoprotein cholesterol (LDL-C).

**Methods:**

Study assessments were conducted at Day 0, 28, 56, 70, 84, 112, and 140, and weight and blood pressure (BP) were measured and fasting blood was collected for analysis of plasma lipids. A DEXA scan was performed and body mass index recorded on Day 0, 70, and 140. Blood glucose, inflammatory, and thyroid markers were measured on Day 0 and 140. Compliance was assessed using weekly 3-day food records and daily blood glucose and ketone monitoring.

**Results:**

The results showed that body fat percentage decreased by 2.25% and 4.41% at Day 70 and 140, respectively (*P* ≤ 0.012). Significant reductions in android, gynoid, and android/gynoid fat ratio and increases in muscle mass occurred by Day 70 and 140. Total cholesterol, LDL-C, and high-density lipoprotein cholesterol were increased and systolic BP and glycated hemoglobin (HbA1c) were decreased at Day 140 (*P* < 0.05). Following this VLCKD for 140 days was found to be safe and was well tolerated.

**Conclusion:**

The VLCKD showed beneficial changes in body composition and cardiometabolic markers in eutrophic and overweight participants in a 140-day study suggesting a future role for this diet in populations at cardiovascular disease risk. Future research with larger sample size in a randomized double blind clinical trial is warranted to confirm these results.

Clinical Trial Registration number: NCT04195594.

## Introduction

Carbohydrate restrictive diets may provide a maximum of 26% calories from carbohydrates and have been shown to have greater efficacy in weight loss and improvements in lipid profile versus low-fat or hypocaloric diets.^1,2^ The ketogenic diet is a very low-carbohydrate diet, generally providing ≤10% calories from carbohydrates. Ketogenic diets for weight loss have been gaining popularity^3^ with the typical ratio of fats to carbohydrates and proteins ranging from 3:1 to 4:1^4^; the goal being to induce “physiological ketosis” marked by increases in blood ketone levels.^5^

One concern with such diets is their conflict with globally accepted dietary guidelines advocating a high-carbohydrate, low-fat dietary pattern.^6^ Current dietary guidelines limit intake of saturated fat largely due to associated increases in low-density lipoprotein cholesterol (LDL-C) concentrations and cardiovascular disease (CVD) risk.^7^ Meta-analyses and reviews conclude the absence of a clear link between saturated fat intake and CVD risk,^8–10^ and mounting evidence indicates a lack of causality between LDL-C levels and CVD risk.^11–15^

Instead, subclasses and particle size of the LDL profile are suggested as a reliable assessment of CVD risk.^16^ Phenotypes A and B have been identified as LDL-C characteristics of value,^17^ with phenotype B recognized as the atherogenic lipoprotein phenotype.^17,18^ Phenotype B is associated with proatherogenic metabolic changes, including reduced insulin sensitivity and high-density lipoprotein cholesterol (HDL-C) and increased intermediate-density lipoprotein (IDL).^17,19^ A ketogenic diet was shown to induce changes in LDL-C subclasses,^20^ and other beneficial changes to lipids have been reported in healthy populations.^20–22^ Beneficial effects on CVD markers^23,24^ in obese populations and improved glycemic control in type 2 diabetes have been reported.^25^

Currently, there are limited studies examining the effects of a ketogenic diet on eutrophic and overweight populations with mildly elevated LDL-C. This open-label pilot study investigated the efficacy of a very-low-carbohydrate ketogenic diet (VLCKD), known as Nic's Ketogenic Diet, for 140 days on cardiovascular health of adults with above optimal or borderline high LDL-C.^26^ CVD risk was assessed by changes in body composition, weight loss, lipid profile, blood pressure (BP), glucose control, and inflammatory markers. This study will be used to inform future larger clinical trials on the use and generalizability of the VLCKD.

## Materials and Methods

### Study design

Unconditional approval from the Institutional Review Board (IRB Services, Aurora, Ontario, Canada) was received on October 25, 2019 (Pro00039616). The open-label pilot study was performed according to the ethical guidelines in the Declaration of Helsinki (2008) and complied with ICH guidelines and Good Clinical Practice (June 10, 1996). The trial was registered at Clinicaltrials.gov

The study was conducted at the KGK Science, Inc., (London, Ontario, Canada) from December 6, 2019 to October 13, 2020. Written informed consent was obtained from all participants before study procedures were being initiated.

### Participants

Participant inclusions were as follows: males and females ages of 30 to 55, body mass index (BMI) 20.0–29.9 kg/m^2^, LDL-C levels between 2.5 and 4.1 mmol/L, and deemed healthy by medical history and laboratory results by the Medical Director (MD). Participants were required to maintain current level of physical activity during the study.

Exclusions were as follows: unable to give informed consent; women who were pregnant, breastfeeding, or planning to become pregnant, menopausal, postmenopausal; following a diet; diabetes or hypertension; used hypertensive medication; a significant major cardiovascular event in the past 6 months assessed by the MD; kidney or liver diseases assessed by the MD; corticosteroids, opioid pain medications, beta-blockers and BP medications, statins, nonsteroidal anti-inflammatory drugs; supplements known to affect weight and/or fat loss or lipid metabolism were considered after a washout period; clinically significant physical examination or abnormal laboratory results; or had any other active or unstable medical condition, that, in the opinion of the MD, may have adversely affected the participant's ability to complete the study or may have posed significant risk.

### Dietary intervention

Participants followed a VLCKD, known as Nic's Ketogenic Diet, for 140 days. The diet provided 5% calories from carbohydrates, 70% from fat, and 25% from protein. Daily calorie needs were assessed by a Registered Dietitian (RD) using the Harris–Benedict equation to calculate basal energy expenditure which adjusts for age, height, weight, gender, and activity level.^27^ This estimate was then multiplied by 0.9 to calculate 90% of their energy requirements for weight maintenance.

Detailed dietary information to participants is provided in Supplementary File S1. Sweeteners, all fruits with the exception of berries, starchy vegetables, low-fat dairy products, soy, grains, beans, and alcohol were to be avoided. Participants were advised to consume green leafy and cruciferous vegetables, avocado, eggs, meat, seafood, fermented foods, high fat dairy products, and organic and grass-fed sources of fats and oils. Mindful eating, which involved eating without distractions and to stop when full, and intermittent fasting were encouraged.

### Study procedures

Full study procedures are provided in Supplementary File S2. Briefly, at screening, participants' medical history and eligibility was reviewed and blood collected. Participants completed a 3-day food record (3DFR) before baseline (Day 0). At baseline, participants received counseling by the RD on the intervention and were provided with an accompanying e-book.

Study outcomes were assessed at Day 0, 28, 56, 70, 84, 112, and 140. At all visits, weight and BP were measured, and fasting blood was collected for plasma lipids. Dual-energy X-ray absorptiometry (DEXA) (Lunar Prodigy Advance, GE Health care) was performed and BMI calculated on Day 0, 70, and 140 and on Day 0 and 140 blood sampled for glucose, glycated hemoglobin (HbA1c), C-reactive protein (CRP), free triiodothyronine (T3), and erythrocyte sedimentation rate (ESR) and safety. Study diaries were dispensed at baseline to record daily sleep and physical activity levels, changes in concomitant therapies, and adverse events (AEs).

Participants monitored blood glucose and ketone levels daily *via* glucose and ketone monitor (KetoMojo) and completed 3DFR and received nutrition counseling as described below. Blood samples were analyzed by a central laboratory (Dynacare, London, ON, Canada).

### Nutrition counseling and compliance

Participants were counseled by an RD on the diet, including the guidelines, the Handy Guide for Food Servings, and accompanying e-book. Information on the ketogenic diet, a summary of e-book, and mini e-course were provided as reference tools.

An online application called Libro, by Nutritics (Dublin, Ireland, United Kingdom), was used to complete 3DFR and ensure study compliance. Participants received a follow-up email 2 weeks after commencement of the diet to ensure that they understood the dietary requirements and to address any questions. Participants' 3DFR were reviewed weekly, and phone and email counseling provided with suggestions on how to comply if 3DFR reflected noncompliance. Compliance was determined using 3DFR and daily ketone and glucose recordings before the first meal of the day.

### Outcomes

The primary outcome of this study was the pre- to postintervention change in body composition (total body fat (%), android fat (%), gynoid fat (%), android/gynoid fat ratio, and muscle mass (%)) by DEXA. The secondary outcomes were the change pre- to postintervention in ESR, CRP, HbA1c, fasting glucose, and T3 after 140 days. The change in weight, BP, and lipid panel was assessed at Day 28, 56, 70, 84, 112, and 140. Safety outcomes were assessed by incidence of AEs, electrocardiogram (ECG), heart rate, clinical chemistry, and hematology.

### Adverse events

AEs were classified based on the description, duration, intensity, frequency, and outcome. All AEs were assessed by the MD for causality and intensity. The Medical Dictionary for Regulatory Activities (MEDRA) terminology version 22.0 was used for coding.

### Statistical analyses

As this was a pilot study, no formal sample size calculation was performed. Primary and secondary end points were analyzed as continuous variables. Continuous repeated measure end points were evaluated using Mixed Model for Repeated Measures; each model included visit timepoint as a fixed effect and baseline measurement as a covariate. Nominals were tabulated as counts and percentages. Missing data for the primary and secondary end points were imputed using the last-observation-carried-forward method or multiple imputation as a sensitivity analysis for the intent-to-treat (ITT) population, and no imputation was performed for the safety population. The ITT and safety populations consisted of all participants who were randomized and for whom postrandomization safety information was available.

Since co-primary outcomes were presented as a real-life global assessment of weight loss and cardiovascular risk, no adjustments for multiplicity were made. Statistical analyses were two-tailed unless otherwise stated. Normality for change in continuous outcomes was assessed using the Shapiro–Wilk test with a p-value threshold of *P* ≥ 0.01, and paired *t*-test or Wilcoxon signed-rank test was used accordingly. Analyses were completed using R (version 3.6.3) with a significance level of 0.05.

## Results

### Study population

**FIG. 1.**
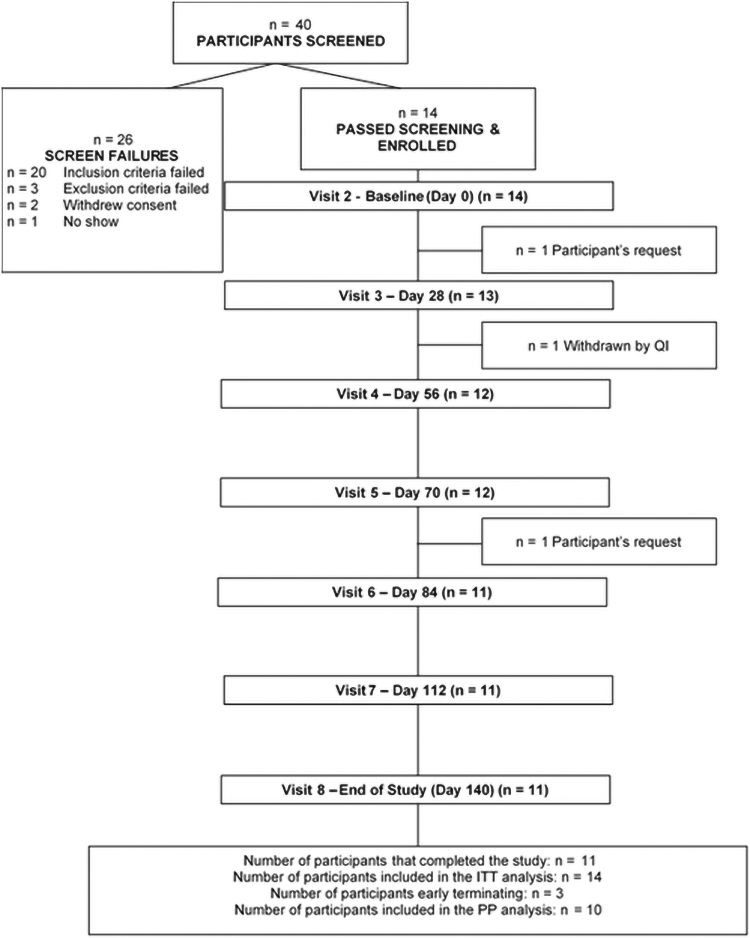
Disposition of study participants.

A total of 40 volunteers were screened, and 14 eligible participants were enrolled (Fig. 1).

Eleven participants completed the study. Participant demographic and lifestyle characteristics are presented in Table 1. Participants ranged from 30 to 53 years old, 50% female, and 71.5% were European. There were no participants who were current smokers, and two former smokers, with 71.4% consuming alcohol weekly or occasionally.

**Table 1.** Participant Baseline Demographic Characteristics and Clinical Measurements (*n* = 14)

Parameter	
Age (years) [Mean ± SD]	39.86 ± 7.36
Gender [*n* (% female)]	7 (50.00%)
Alcohol use [*n* (%)]	
None	4 (28.60%)
Occasionally	5 (35.70%)
Weekly	5 (35.70%)
Tobacco use [*n* (%)]	
Ex-smoker	2 (14.30%)
No	12 (85.70%)
Weight (kg) [Mean ± SD]	76.49 ± 10.80
BMI (kg/m^2^) [Mean ± SD]	25.92 ± 2.39
SBP (mmHg) [Mean ± SD]	118.50 ± 5.28
DBP (mmHg) [Mean ± SD]	77.04 ± 9.24
Heart rate (bpm) [Mean ± SD]	70.88 ± 10.33

BMI, body mass index; DBP, diastolic blood pressure; *n*, number of participants; SBP, systolic blood pressure; SD, standard deviation.

### Body composition and body weight

**FIG. 2.**
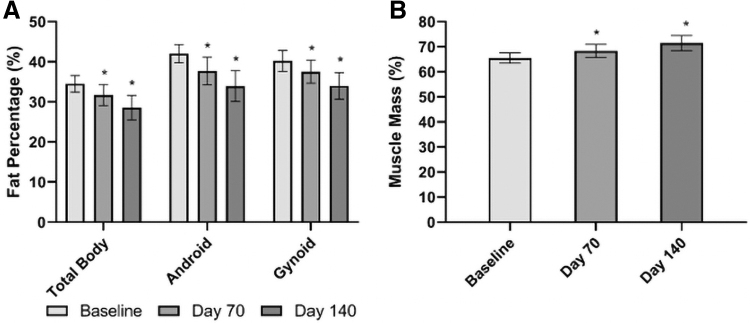
Mean total body, android, and gynoid fat percentage **(A)** and muscle mass percentage **(B)** of participants at baseline and Day 70 and 140 of following a VLCKD. * indicates significant change from baseline (*P* < 0.05). VLCKD, very-low-carbohydrate ketogenic diet.

There was a significant 2.25% and 4.41% reduction in body fat percentage from baseline at Day 70 and 140, respectively (Fig. 2A). Android and gynoid fat percentages were significantly reduced, resulting in android/gynoid fat ratio reductions (*P* ≤ 0.042). There was a 2.24% and 4.38% increase in muscle mass at Day 70 and 140, respectively (*P* ≤ 0.012) (Fig. 2B).

**FIG. 3.**
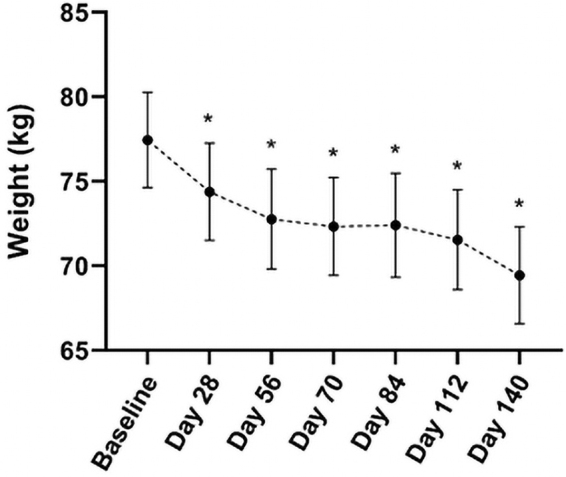
Mean body weight from baseline to Day 140 of participants following a VLCKD. Values presented as mean ± SEM; * indicates *P* < 0.001. SEM, standard error of the mean.

A significant reduction in body weight was observed at Day 28, 56, 70, 84, and 112. After 56 days of the VLCKD, there was a significant 5.65% reduction in weight. This weight loss was maintained to Day 140, resulting in a 10.65% reduction from baseline (Fig. 3). All participants had a reduction in weight at Day 70, 84, 112, and 140.

There was a 6.2% and 10.6% reduction in BMI at Day 70 and 140, respectively (*P* < 0.001) (Table 2). Mean BMI changed from an overweight BMI categorization of 26.27 kg/m^2^ at baseline to normal weight of 23.60 and 23.31 kg/m^2^ at Day 70 and 140, respectively.

**Table 2.** Body Mass Index of Participants at Baseline and Day 70 and 140 of Following a Very-Low-Carbohydrate Ketogenic Diet

	Mean ± SD	*P*
Day 0	26.27 ± 2.2	
Day 70	24.41 ± 2.08	
Day 140	23.31 ± 2.18	
Change from Day 0 to 70	−1.64 ± 0.83	<0.001
Change from Day 0 to 140	−2.82 ± 1.49	<0.001

### Lipid profile parameters

**FIG. 4.**
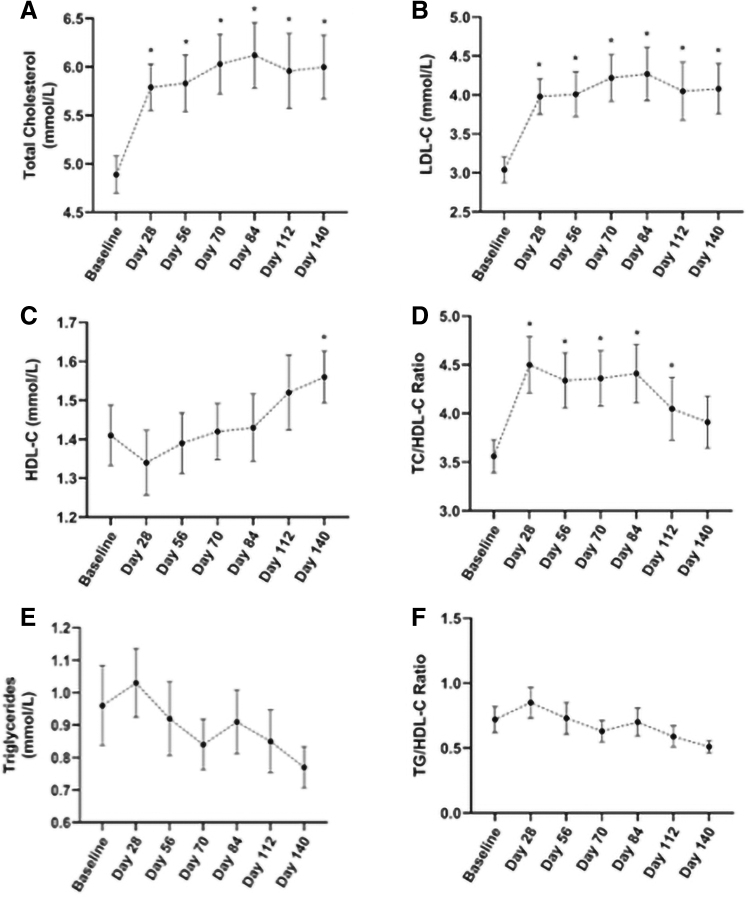
Mean TC **(A)**, LDL-C **(B)**, HDL-C **(C)**, TC/HDL-C ratio **(D)**, triglycerides **(E)**, and triglyceride/HDL-C ratio **(F)** from baseline to Day 140 of participants following a VLCKD. Values presented as mean ± SEM; * indicates *P* < 0.05. HDL-C, high-density lipoprotein cholesterol; LDL-C, low-density lipoprotein cholesterol; TC, total cholesterol.

Total cholesterol (TC) increased from baseline at Day 28, 56, 70, 84, 112, and 140 (*P* ≤ 0.006) (Fig. 4A). There were significant increases in LDL-C levels at all visits (Fig. 4B); however, these levels stayed within the acceptable laboratory range for this population and were not considered to be clinically relevant. There was a 12.8% increase in HDL-C (*P* = 0.011) (Fig. 4C), with 73% of participants having increased HDL-C at Day 140. TC/HDL-C ratio was increased at Day 28, 56, 70, 84, and 112 (*P* ≤ 0.018); however, there was no significant difference at Day 140 (Fig. 4D). There was no change in TG level or TG/HDL-C ratio during the study (Fig. 4E, F).

Ninety-three percent of participants had TC levels outside the laboratory range at one or more study visits. Of these participants, five participants had TC levels considered as high.^26^ These high TC levels appear to begin at Day 28 or 56 except for one participant with high TC at baseline. All other lipid parameters, including LDL-C, HDL-C, and TG, remained within the normal range except for one participant whose HDL-C level was below the normal range at Day 28 and 84. All excursions outside the range were deemed not clinically relevant and did not require additional management.

### BP and metabolic parameters

**FIG. 5.**
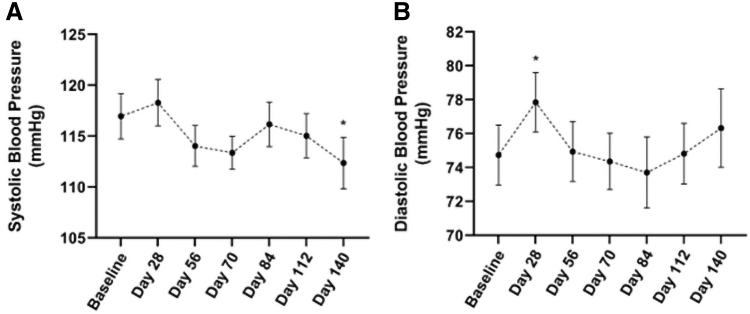
Mean systolic **(A)** and diastolic **(B)** blood pressure from baseline to Day 140 of participants following a VLCKD. Values presented as mean ± SEM; * indicates *P* < 0.05.

Systolic BP was reduced by 5.3% from baseline at Day 140 of the VLCKD (*P* = 0.019) (Fig. 5). There was a significant increase in DBP at Day 28; however, no significant changes at Day 56, 70, 84, 112, and 140.

There was a reduction in HbA1c and thyroid hormone T3 at Day 140 (*P* ≤ 0.003) (Table 3). There was no significant change in fasting glucose, ESR, or CRP.

**Table 3.** Inflammatory, Glucose Control, and Thyroid Markers of Participants at Baseline and Day 70 and 140 of Following a Very-Low-Carbohydrate Ketogenic Diet

	Mean ± SD	*P*
CRP		
Day 0	2.05 ± 2.55	
Day 140	1.24 ± 1.16	
Change from Day 0 to 140	−0.71 ± 2.10	0.610
ESR		
Day 0	7.79 ± 7.23	
Day 140	7.55 ± 5.16	
Change from Day 0 to 140	0.36 ± 4.43	0.531
HbA1c		
Day 0	5.40 ± 0.36	
Day 140	5.25 ± 0.34	
Change from Day 0 to 140	−0.22 ± 0.18	0.003
Fasting glucose		
Day 0	5.07 ± 0.38	
Day 140	5.01 ± 0.62	
Change from Day 0 to 140	−0.06 ± 0.37	0.626
T3		
Day 0	5.40 ± 0.69	
Day 140	4.56 ± 0.76	
Change from Day 0 to 140	−0.85 ± 0.55	<0.001

CRP, C-reactive protein; ESR, erythrocyte sedimentation rate; HbA1c, glycated hemoglobin; T3, free Triiodothyronine.

### Safety evaluation

A total of 44 postemergent AEs were reported in this study. There were 13 AEs categorized as possibly related to the diet reported by five participants. Of these, eight were related to gastrointestinal issues and included constipation, diarrhea, heartburn, nausea, and vomiting and were reported by four participants. All AEs were resolved by Day 140.

Hematology, clinical chemistry, electrolytes, and liver and kidney function markers remained within healthy clinical reference ranges (data not shown). Markers out of laboratory range were deemed not clinically significant, and all participants were determined as healthy when exiting the study. There were no clinically relevant ECG or HR findings (data not shown).

### Diet composition and compliance

Based on 3DFR, there was a significant daily calorie reduction of 594 kcal from preintervention to Day 140 (Table 4). Carbohydrate intake was significantly reduced and fat significantly increased during all weeks of the study. Daily macronutrient composition ranged from 5.28% to 7.42% of calories from carbohydrates, 69.42% to 72.18% from fat, and 22.54% to 23.07% from protein (Table 4). This macronutrient composition aligned with the VLCKD requirement of 5% calories from carbohydrate, 70% from fat, and 25% from protein confirming compliance during the 140 days.

**Table 4.** Energy, Macronutrient, and Specific Types of Dietary Fat Reported by Participants Before Beginning the Very-Low-Carbohydrate Ketogenic Diet (Week 0) and After 140 Days of the VLCKD (Week 20) Assessed by 3-Day Food Records

	Mean ± SD	*P*
Energy (kcal/day)		
Week 0	2206.63 ± 592.38	
Week 20	1755.83 ± 539.39	
Change from Week 0 to 20	−594.14 ± 794.19	0.043
Carbohydrate (g/day)		
Week 0	196.41 ± 70.21	
Week 20	14.86 ± 3.90	
Change from Week 0 to 20	−163.28 ± 75.40	<0.001
Protein (g/day)		
Week 0	99.47 ± 44.76	
Week 20	99.82 ± 29.20	
Change from Week 0 to 20	−15.31 ± 51.11	0.341
Total fat (g/day)		
Week 0	107.63 ± 41.66	
Week 20	141.21 ± 46.60	
Change from Week 0 to 20	16.95 ± 48.38	0.014
Saturated fat (g/day)		
Week 0	38.44 ± 13.87	
Week 20	59.58 ± 22.65	
Change from Week 0 to 20	15.43 ± 19.73	0.001
Monounsaturated fat (g/day)		
Week 0	25.59 ± 17.18	
Week 20	40.64 ± 16.51	
Change from Week 0 to 20	11.38 ± 17.90	0.021
Polyunsaturated fat (g/day)		
Week 0	12.51 ± 10.10	
Week 20	12.35 ± 5.10	
Change from Week 0 to 20	−1.94 ± 8.12	0.979
Polyunsaturated fat (g/day)		
Week 0	1.06 ± 0.92	
Week 20	1.47 ± 1.05	
Change from Week 0 to 20	0.51 ± 1.14	0.184

VLCKD, very-low-carbohydrate ketogenic diet.

Mean ketone concentrations at preintervention were 0.52 mmol/L and reached 0.81 mmol/L on Day 140 with the VLCKD (data not shown). Glucose ketone index, a measure of ketosis based on the relationship between fasting glucose and ketone concentrations, was 13.77 at prior to intervention and 8.78 on Day 140.

## Discussion

After 140 days on the VLCKD participants showed a 4.41% reduction in body fat, which is comparable to the results reported by other studies on ketogenic or low-carbohydrate diets of similar duration.^23,28^ These results were supported by the decrease in the android/gynoid fat ratio indicating a positive change in body fat distribution and decreased risk of metabolic syndrome.^29,30^ This ratio is more highly correlated with cardiometabolic dysregulation than BMI or android or gynoid fat percentage alone,^31^ suggesting that this reduction is clinically relevant in addressing cardiometabolic disease risk. After 56 days, there was a clinically relevant 5.65% reduction in weight,^32^ which was maintained to the end of study. This resulted in a 10.65% reduction in weight by Day 140 and within the range of 2.1–13.1 kg reported by other VLCKDs.^33–38^

There was an associated 4.38% increase in muscle mass indicating that weight loss was attributed to fat loss rather than muscle mass during the 140-day intervention period.

The decrease in T3 levels perhaps influenced the LDL-C increase seen in the participants, as T3 is involved in LDL-C particle clearance.^39,40^ Furthermore, dietary cholesterol was increased by 303.37 mg/d, exceeding recommendations,^41^ and may have contributed to a rise in blood cholesterol levels.^20^ Research suggests that blood cholesterol can be influenced by a variety of factors, including genetics, hormones, and obesity.^42^ It has been suggested that LDL-C profile, including subclasses and particle size rather than total levels, may determine atherogenic phenotypes A and B^17^ and CVD risk.^16^ The current study did not examine LDL-C subclasses; however, estimation of phenotype may be achieved by examining cutoffs for low HDL-C and high TG and TG/HDL-C ratio^43–45^ based on the strong association between LDL-C profile, HDL-C, and TG levels.

A significant 12.8% increase in HDL-C is clinically relevant as a 1% increase in HDL-C is associated with a 2%–3% reduction in CVD risk.^46^ The 0.23 mmol/L TG reduction together with HDL-C changes may have stronger implications for CVD risk as they are associated with LDL-C phenotype. At Day 140, all participants had a lipid profile suggesting a nonatherogenic phenotype A. This shift occurred primarily as a result of high HDL-C (≥1.5 mmol/L) and low TG/HDL-C ratio (≤0.8).^43^

Increased consumption of monounsaturated fat is believed to improve insulin sensitivity.^47^ Therefore, it is possible that increasing intake of monounsaturated fat through consumption of avocado, macadamia, and olive oil, as part of the VLCKD, may have contributed to the 4.0% improvement in HbA1c. Of further clinical relevance was the 6.52 mmHg reduction in SBP from baseline at Day 140 as a 2 mmHg reduction in BP is associated with reduced CVD risk.^48,49^

This study was limited by the fact that it was an open label design and does not allow for comparison against a group not receiving the intervention.^50^ Placebo effects in diet studies can be influenced by participant expectation, personal beliefs, taste preferences, and previous exposure^50^ influencing quantitative markers,^51^ and successful interventions vary due to personal preferences and metabolic differences.^52^ Future studies are warranted with this VLCKD compared with other interventions such as low-carbohydrate or low-fat diets.^1,2^ The small sample size and specific population chosen for this investigation restrict the ability to make reasonable conclusions, as well as generalizability to other populations that are at CVD risk.^53^ Therefore larger studies with a more diverse population should be conducted to understand the application of VLCKD on other populations.

This study did not measure LDL-C subclasses or particle size; therefore, conclusions related to changes in atherogenic phenotype cannot be made and should be explored in future studies. Furthermore, the increases in LDL-C in this study require further evaluation in long term studies in relation to individual cardiovascular risk.

Participants had a 594.14 daily calorie reduction by the end of study which is comparable to the caloric reduction with a 6-month low-carbohydrate diet despite not restricting energy intake.^52^ A reduction in hunger has been previously reported with ketogenic diets,^39,54^ suggesting that the unintentional calorie reduction is due to improved satiety. Calorie reduction and substantial weight loss may have confounded the changes in cardiometabolic markers, including HDL-C, TG, HbA1c, and SBP, in this study. Maximum weight loss with a ketogenic diet has been proposed to be achieved by 6 months with improvement of cardiometabolic markers continuing up to 24 months.^52^ It is possible that with longer study duration, improvements in HbA1c, lipid profile, and BP observed in the current study may have continued, after the weight loss period had subsided. Future studies should explore the efficacy of VLCKD over a period up to 2 years to examine the effect on cardiometabolic markers independent of weight loss.

There have been few longer term studies examining ketogenic diets among obese participants,^24,55^ with similar limitations of open-label design and lacking a control group. While the findings of this study suggest beneficial changes in cardiometabolic outcomes, the potential detrimental effects of a long-term VLCKD need to be recognized. This may include acidosis, hyperuricemia, dehydration, and gastrointestinal issues such as constipation and nausea.^4^

There is a dearth of literature regarding the effect of a VLCKD on the gut microbiota^56,57^ particularly for periods greater than 4 months.^58^ There is also concern related to creating micronutrient deficiencies, which is often addressed by providing multivitamin supplements.^55,59^ Several studies have used a ketogenic diet effectively for shorter periods of time, followed by a weight maintenance diet for 12^59^ to 24 months.^52^ This may be a useful approach in mitigating potential detrimental effects while providing the cardiometabolic effects, particularly in populations with increased cardiovascular risk. Future studies should also examine the adjustments that are made after a VLCKD and return to habitual diet as this has not been previously examined.

## Conclusion

This open-label study demonstrated that 140 days of a VLCKD, known as Nic's Ketogenic Diet, significantly reduced body fat, weight, BMI, SBP, and HbA1c and increased muscle mass in healthy participants with mildly elevated LDL-C levels. Significant increases in TC, LDL-C, and HDL-C were observed after 140 days which may have been driven by a variety of factors, including diet, genetics, and weight loss. This diet was found to be safe as assessed by AEs, clinical chemistry, hematology, heart rate, and ECG. The beneficial changes in body composition and clinically relevant changes in cardiometabolic markers suggest that VLCKD could possibly be used as a strategy to mitigate CVD risk; however, these findings need to be explored in future randomized clinical trials.

## Supplementary Material

Supplemental data

Supplemental data
