# Supplementary material for: Examining the Efficacy of a Very-Low-Carbohydrate Ketogenic Diet on Cardiovascular Health in Adults with Mildly Elevated Low-Density Lipoprotein Cholesterol in an Open-Label Pilot Study
Source: Metab Syndr Relat Disord. 2022 Mar 15;20(2):94–103. doi: 10.1089/met.2021.0042 (PMC8972001; doi:10.1089/met.2021.0042)
Supplement: Supplemental data [file Suppl_FileS1.docx]

Supplementary File 1. Dietary guidelines for the very-low-carbohydrate ketogenic diet provided to participants during the study

**Dietary Guidelines**

Please use the following guidelines to help you make meal and snack choices between study visits that are appropriate for the study.

**Foods to Avoid**

- All sweeteners other than Erythritol and Stevia
- Soybeans and soy products (tofu, tempeh, natto, edamame, soy milk, etc.)
- Grains
- Beans, peas and pulses
- Protein bars, shakes, and supplements
- Starchy vegetables (potato, yam, carrot, etc.)
- Low-fat dairy products
- Alcohol except spirits
- All fruits except berries listed below

**Foods/Items to Limit**

- Alcohol (no more than 2 standard servings of liquor/spirits per day)
  - Wine, beer, cocktails, coolers, ciders are all **not permitted**
  - Permitted: vodka, gin, whiskey, etc.
- Broccoli, cabbage, brussels sprouts
- Fruit such as strawberries, blueberries, and raspberries

**Suggested Foods:**

- Vegetables: *Green leafy and cruciferous vegetables.*
  - Lettuce
  - Arugula
  - Spinach
  - Mushrooms
  - Asparagus
- Bok choy
- Kale
- Collard greens
- Meats: *Fattier sources of protein are encouraged. Watch out for added sugars.*
  - **Fish:** Wild caught fish such as catfish, cod, flounder, halibut, mackerel, mahi-mahi, salmon, snapper, trout, and tuna.
  - **Shellfish:** Clams, oysters, lobster, crab, scallops, mussels, and squid.
  - **Beef:** Ground beef, steak, roasts, and stew meat.
  - **Pork:** Ground pork, pork loin, pork chops, tenderloin, and ham.
  - **Poultry:** Chicken, duck, quail, pheasant and other wild game.
  - **Organ meats:** Heart, liver, kidney, and tongue.
  - **Other Meats:** Veal, Goat, Lamb, Turkey and all kinds of wild game.
  - **Bacon and Sausage:** Check labels and avoid anything cured in sugar or containing artificial fillers.
- Fats and Oils: *Organic and grass-fed sources are always best.*
- Lard
- Tallow
- Butter
- Macadamia/Brazil Nuts
- Butter/Ghee
- Mayonnaise
- Coconut Butter
- Cocoa Butter
- Olive Oil
- Coconut Oil
- Avocado Oil
- Macadamia Oil
- MCT Oil
  - - Eggs
    - Fermented foods (sauerkraut, kimchi, lacto-fermented vegetables, etc.)
    - Avocado
    - High fat dairy (cheese, heavy cream, butter, etc.)
