# Supplementary material for: Examining the Efficacy of a Very-Low-Carbohydrate Ketogenic Diet on Cardiovascular Health in Adults with Mildly Elevated Low-Density Lipoprotein Cholesterol in an Open-Label Pilot Study
Source: Metab Syndr Relat Disord. 2022 Mar 15;20(2):94–103. doi: 10.1089/met.2021.0042 (PMC8972001; doi:10.1089/met.2021.0042)
Supplement: Supplemental data [file Suppl_FileS2.docx]

Supplementary File 2. Detailed study procedures

1. **Height and Weight**

Weight measurements were performed with shoes removed and bladder empty on calibrated scales at all visits.

At least two separate measurements were taken at each visit. If the two measurements were more than 0.5 kg (1.1 lbs.) apart, a third measurement was taken. The two closest values were selected and entered in the database.

Measurement of height was with the participant’s shoes removed. The participant’s knees were straightened, and head held upright.

1. **Blood Pressure and Heart Rate**

In-office, seated, resting blood pressure assessment:

The participant was seated comfortably with their back supported and the upper arm bared without restrictive clothing. Feet were flat on the floor and legs were not be crossed. The participant rested in this position for at least 5 minutes prior to the first reading.

At screening:

Seated blood pressure was checked in both arms and if different, the arm with the higher systolic blood pressure reading was taken for measurements. If the systolic blood pressure was the same in both arms, the arm with the higher diastolic blood pressure was used. If both were equal, then the left arm was used. In the chosen arm, a second measurement was taken at least 1 minute from the first measurement. If a difference of more than 8 mmHg existed between the two readings a third reading was taken. An average of the two lowest readings from the chosen arm were taken for the determination of inclusion into the study. Per the QI’s opinion, a high office blood pressure was rechecked manually after the participant was given a glass of water and was seated for 15 min. Also, the participant was queried about their usual blood pressure.

The arm chosen for use at the initial visit was documented in the study file and used in all subsequent visits. Once enrolled in the study, BP was be measured in the chosen arm. Three readings were made, averaged and recorded.

Heart Rate (beats/min) was measured using the reading on the automated blood pressure monitor, or manually by the clinical coordinator placing their index finger on the participant's radial artery while observing a timer and counting the number of beats over 30 seconds and then multiplying the number by two. This was repeated for a total of three measurements.

1. **Blood Sample Collection and Analysis**

At all study visits, a phlebotomist performed the venipuncture procedure to collect the necessary blood samples. Participants were placed in a comfortable seated position with their desired arm, at the phlebotomist’s discretion, fully extended and supported with a pillow. A tourniquet was applied 3-4 inches above the elbow with the participants opening and closing their fist a few times to allow the phlebotomist to manually determine the approximate size, depth, and location of the vein. Following the site of the venipuncture being appropriately sterilized, the phlebotomist collected the sample using the vacutainer system according to the relevant laboratory order requirement. Once collection was complete a cotton ball was immediately placed on the venipuncture site which was be periodically checked to ensure clotting has begun at which point a clean cotton ball was applied and secured with tape.

All blood measurements were analyzed by diagnostic laboratory, Dynacare (London, ON, Canada), using standard procedures. The lipid panel, consisting of triglycerides (TG), total cholesterol (TC), LDL-C and HDL-C, was analyzed by enzymatic colorimetric assay from blood collected at Day 0, 28, 56, 70, 84, 112 and 140. C-reactive protein (CRP), free triiodothyronine (T3), glycated hemoglobin (HbA1c), fasting glucose and erythrocyte sedimentation rate (ESR) were analyzed from blood drawn at Day 0 and 140. C-reactive protein (CRP) was analyzed by particle enhanced immunoturbidimetric assay with an analytical range of 0.3 – 350 mg/L and T3 was analyzed by electrochemiluminescence immunoassay (ECLIA) with an analytical range of 0.3 - 10.0 nmol/L. Turbidimetric inhibition immunoassay (TINIA) was used to analyze HbA1c and optical density of microagglutination was used to measure ESR with an analytical range of 2 – 120 mm/hr.

The safety outcomes were analyzed from blood drawn at screening and Day 140. Hematology (WBC count with differential, RBC count, hemoglobin, hematocrit, platelet count, RBC indices (MCV, MCH, MCHC, RDW, MPV)) were measured by Coulter Principle. Liver function was assessed by ALT, AST and total bilirubin using enzymatic spectrophotometric and Roche Colorimetric diazo method, respectively. Kidney function was assessed based on creatinine, electrolytes (Na, K, Cl), and eGFR. Creatinine was measured by enzymatic assay, electrolytes were measured by ion-selective electrode (ISE) and eGFR was calculated using the Chronic Kidney Disease Epidemiology Collaboration (CKD-EPI) equation.

Urine pregnancy tests were conducted at the KGK Clinic for participants of childbearing capacity at screening, Day 0 and Day 140.

1. **Electrocardiogram (ECG)**

The participant electrode-skin area was prepared for ECG by the clinic coordinator at the KGK clinic (London, ON, Canada) to ensure a good electrode contact. The electrode was not be placed over bones, irritated skin, areas where there was a lot of muscle movement or incisions. The participant was instructed to breathe calmly and not read or talk and have no metal contacts including belt, keys and coins during the ECG test.

1. **Dual Energy X-Ray Absorptiometry (DXA) Scan**

The DXA scan (Lunar Prodigy Advance, GE Healthcare) was performed at the KGK clinic (London, ON, Canada) at Day 0, 70 and 140 by trained technicians. The DXA scan is a form of X-ray radiation that measures the body tissue density which will be converted and used for assessment of the study outcomes. Coordinators reminded participants 24 hours before the day of the DXA assessment for the necessary instructions prior to the scan.
